# Dataset of cathepsin L-like CP inhibition of *Naegleria fowleri* and *Acanthamoeba castellanii* by ppTvCP4r from *Trichomonas vaginalis*

**DOI:** 10.1016/j.dib.2018.03.029

**Published:** 2018-03-13

**Authors:** Rosa E. Cárdenas-Guerra, Moisés Martínez-Castillo, Jaime Ortega-López, Mineko Shibayama, Rossana Arroyo

**Affiliations:** a Departamento de Infectómica y Patogénesis Molecular, Centro de Investigación y de Estudios Avanzados del Instituto Politécnico Nacional (CINVESTAV-IPN), Av. IPN # 2508, Col. San Pedro Zacatenco, Delg. Gustavo A. Madero, CP 07360 Ciudad de México, Mexico; b Departamento de Biotecnología y Bioingeniería, Centro de Investigación y de Estudios Avanzados del Instituto Politécnico Nacional (CINVESTAV-IPN), Av. IPN # 2508, Col. San Pedro Zacatenco, Delg. Gustavo A. Madero, CP 07360 Ciudad de México, Mexico

**Keywords:** *Acanthamoeba castellanii*, Cathepsin L-like CPs, Cysteine proteinase inhibitors, *Naegleria fowleri*, ppTvCP4r, *Trichomonas vaginalis*

## Abstract

The recombinant TvCP4 prepro region (ppTvCP4r) acts as an exogenous inhibitor of cathepsin L-like CPs from *Trichomonas vaginalis* (Cárdenas-Guerra et al., 2015 [1]). Here, we present the dataset of the trichomonad ppTvCP4r inhibitory effect against the CP proteolytic activities from other microorganisms, such as *Naegleria fowleri* and *Acanthamoeba castellanii* free-living amoeba. The proteolytic activity inhibition of total crude extracts (TCEs) of *N. fowleri* and *A. castellanii* was determined and recorded using a fluorogenic substrate specific for cathepsin L CPs without or with a ppTvCP4r treatment at different concentrations and pH.

## Specifications Table

**Table:** 

Subject area	Biochemistry
More specific subject area	Enzyme inhibition
Type of data	Figure and Table
How data was acquired	Fluorescence intensity measured in a Microplate Reader spectrofluorometer at 355 and 460 nm excitation and emission wavelengths.
Data format	Raw, analyzed
Experimental factors	Total crude extracts (TCEs) of *N. fowleri* and *A. castellanii* trophozoites lysed in PBS by freeze-thaw cycles, a fluorogenic substrate for cathepsin L CPs, recombinant ppTvCP4 as CP inhibitor at 25 °C and at different concentrations, time, and pH during inhibition assays.
Experimental features	Inhibitory effect of ppTvCP4r against CP proteolytic activities of *N. fowleri* and *A. castellanii.*
Data source location	CINVESTAV-IPN, Mexico City, Mexico
Data accessibility	The data is available with this article

## Value of the data

• The data show the ability of a fluorogenic substrate to detect low levels of CP proteolytic activity not detectable by zymography.
• The data shows the potent enzyme inhibitory action of the recombinant prepro region of cathepsin L-like CP from *Trichomonas vaginalis* (ppTvCP4r) on CPs from free-living amoeba *Naegleria fowleri* and *Acanthamoeba castellanii.*
• The data shows the potential use of the ppTvCP4r inhibitor to help determine the potential role of CPs in the pathogenesis of this free-living amoeba that could open up its further potential use for drug targeting.
• The data also shows the potential use of *T. vaginalis* ppTvCP4r against cathepsin L-like CPs from other organisms including human pathogens.

## 1 Data

**Fig. 1:**
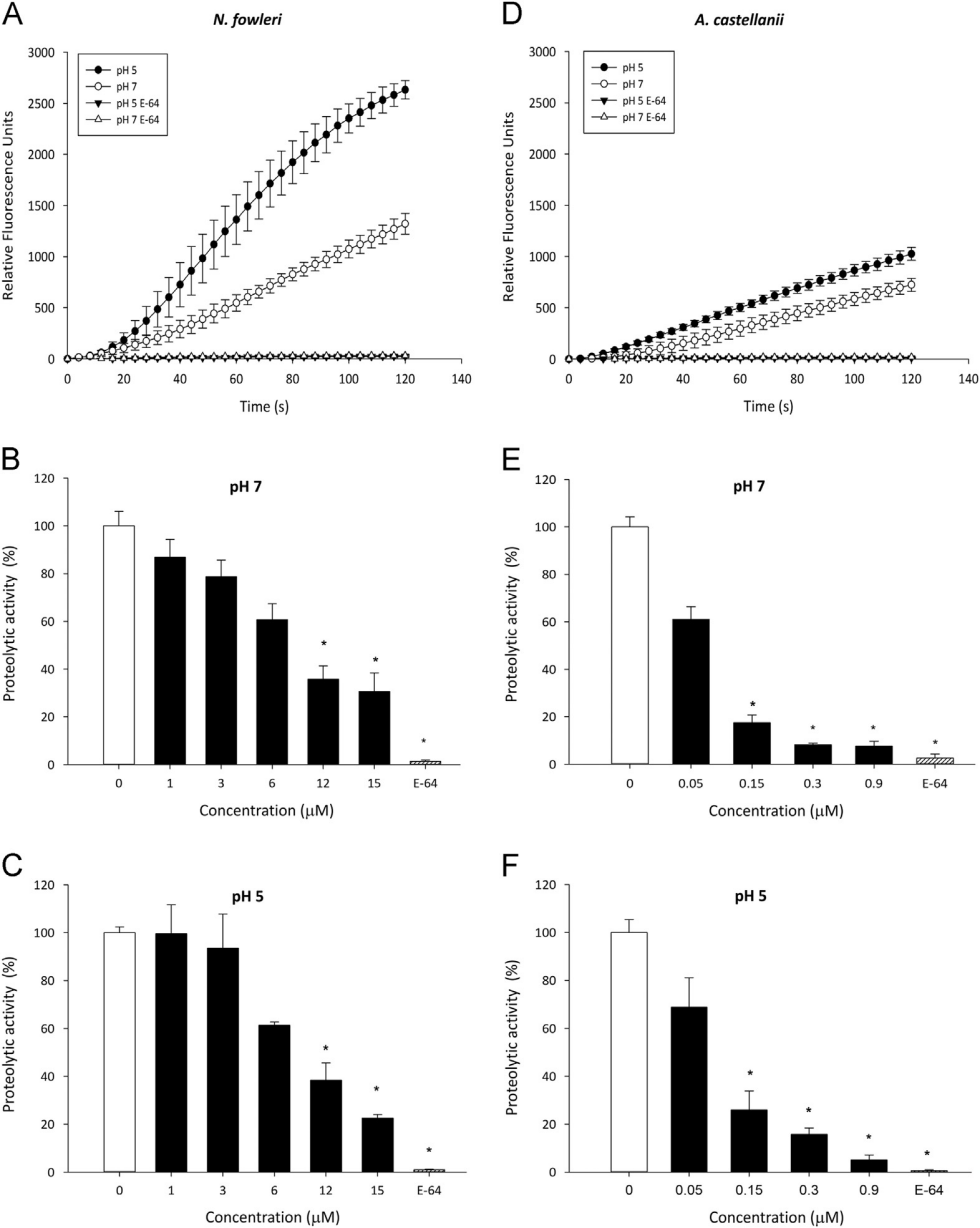
Trichomonad ppTvCP4r inhibitory activity against cathepsin L-like proteases from *Naegleria fowleri* and *Acanthamoeba castellanii* free-living amoeba. **(A, D)** Relative Fluorescence Unit (RFU) as a function of time (s) of *N. fowleri* (**A**) and *A. castellanii* (**D**) total crude extracts (TCEs) at pH 7 (white circles) and pH 5 (black circles) was measured using the fluorogenic substrate (Z-Phe-Arg-AMC) specific for cathepsin L CPs. E-64 (*trans*-Epoxysuccinyl-L-leucylamido(4-guanidino)butane) was used as a positive control for the specific inhibition of the cathepsin L-like CP activities at pH 7 (white triangles) and pH 5 (black triangles). **(B–C, E–F)** Percentage of inhibition of CP proteolytic activity of *N. fowleri* and *A. castellanii* TCEs (%) in the presence of different concentrations of ppTvCP4r (black bars) (1.0–15.0 μM) (**B–C**) and (0.05–0.9 μM) (**E–F**) and 100 μM E-64 (hatched bar) at pH 7 and pH 5. The proteolytic activity without ppTvCP4r treatment (white bars) was taken as 100% in each case (Table 1). The error bars indicate the standard errors of the mean (SEM) of at least three independent experiments in triplicate. Significant differences (*P* < 0.001) between the results are marked with asterisks.

The prepro regions of the cathepsin L-like cysteine proteinases (CPs) are also inhibitors of related peptidases, in which the selectivity correlates with the degree of similarity in the prepro region sequences of the target proteinases (Wiederanders et al., 2003 [2]; Yamamoto et al., 2002 [3]). The development of CP inhibitors has provided useful tools to study and to profile the cellular proteolytic activity, to identify their extra-lysosomal functions in cells and pathogen organisms and for potential application in medicine as candidates for antiparasitic chemotherapy, among other uses (Turk et al., 2002 [4]; Sajid and McKerrow, 2002 [5]). While the inhibitors developed may not necessarily prove useful as drugs because of the disadvantages that present such as bioavailability, low toxicity, and selectivity, they still have immense value as research tools in studying the biological function of targeted enzymes (Dubin, 2005 [6]). The dataset of this article provides information on the ppTvCP4r (trichomonad recombinant prepro region of TvCP4) inhibitory activity against cathepsin L-like proteases from *Naegleria fowleri* and *Acanthamoeba castellanii* free-living amoeba using a specific fluorogenic substrate (Fig. 1 and Table 1). The concentration and time-dependent CP proteolytic activities at pH 5 and 7 in the absence and presence of ppTvCP4r have been recorded and presented.

**Table 1:** Inhibition of the proteolytic activity of *A. castellanii* and *N. fowleri* Total Cell Extract (TCE) by trichomonad ppTvCP4r.

	**ppTvCP4r**	**Enzyme activity ± SD**^b^		
	**(µM)**	**RFU/s**	RFU/(s * µg)	**(%)**
***A. castellanii***^a^				
**pH 7**	0.00	7.23 ± 0.308	0.241 ± 0.010	100.0 ± 4.3
	0.05	4.41 ± 0.387	0.147 ± 0.013	61.0 ± 5.4
	0.15	1.27 ± 0.231	0.042 ± 0.008	17.5 ± 3.2
	0.30	0.59 ± 0.045	0.020 ± 0.002	8.2 ± 0.6
	0.90	0.55 ± 0.153	0.018 ± 0.005	7.6 ± 2.1
	**E-64 (100** **μM; positive control)**	**0.18 ± 0.128**	**0.006 ± 0.004**	**2.5 ± 1.8**
**pH 5**	0.00	9.49 ± 0.514	0.316 ± 0.017	100.0 ± 5.4
	0.05	6.53 ± 1.167	0.218 ± 0.039	68.8 ±12.3
	0.15	2.46 ± 0.757	0.082 ± 0.025	25.9 ± 8.0
	0.30	1.49 ± 0.255	0.050 ± 0.009	15.7 ± 2.7
	0.90	0.48 ± 0.202	0.016 ± 0.007	5.0 ± 2.1
	**E-64 (100** **μM; positive control)**	**0.05 ± 0.036**	**0.002 ± 0.001**	**0.6 ± 0.4**
***N. fowleri***^c^				
**pH 7**	0.0	15.55 ± 0.944	0.239 ± 0.015	100.0 ± 6.0
	1.0	13.50 ± 1.164	0.208 ± 0.018	86.9 ± 7.5
	3.0	12.23 ± 1.081	0.188 ± 0.017	78.7 ± 7.0
	6.0	9.44 ± 1.031	0.145 ± 0.016	60.7 ± 6.6
	12.0	5.55 ± 0.878	0.085 ± 0.014	35.7 ± 5.7
	15.0	4.76 ± 1.206	0.073 ± 0.019	30.6 ± 7.8
	**E-64 (100 μM; positive control)**	**0.21** ± **0.083**	**0.003** ± **0.001**	**1.4** ± **0.5**
**pH 5**	0.0	29.54 ± 0.717	0.454 ± 0.011	100.0 ± 2.4
	1.0	29.41 ± 3.574	0.452 ± 0.055	99.5 ± 12.1
	3.0	27.62 ± 4.211	0.425 ± 0.065	93.5 ± 14.3
	6.0	18.11 ± 0.412	0.279 ± 0.006	61.3 ± 1.4
	12.0	11.34 ± 2.115	0.174 ± 0.033	38.4 ± 7.2
	15.0	6.66 ± 0.439	0.102 ± 0.007	22.5 ± 1.5
	**E-64 (100** **μM; positive control)**	**0.28** ± **0.055**	**0.004** ± **0.001**	**0.95** ± **0.2**

a A total of 30 μg of *A. castellanii* TCE was used in each assay.

b ± SD, standard deviation of each value.

c A total of 65 μg of *N. fowleri* TCE was used in each assays.

## 2 Experimental design, materials, and methods

### 2.1 Enzyme inhibition assays

All enzyme inhibition assays were performed at 25 °C for 120 s using a fluorogenic substrate (Z-Phe-Arg-AMC; Peptide Institute Inc., Osaka, Japan) specific for cathepsin L CPs. The reaction was initiated by the addition of the fluorogenic substrate into the reaction wells of a 96-well plate containing total crude extracts (TCEs). The increase in fluorescence intensity due to the release of aminomethyl coumarin (AMC) was measured using a Gemini EM Microplate Reader spectrofluorometer (SpectraMaxR Gemini EM; Molecular Devices, Sunnyvale, CA, USA) at 355 and 460 nm excitation and emission wavelengths, respectively. For all proteolytic activity inhibition assays, the kinetics were obtained using different concentrations (1, 3, 6, 12, and 15 μM) of the recombinant protein ppTvCP4 (ppTvCP4r) [1] over a TCE of *N. fowleri* (65 μg) and (0.05, 0.15, 0.3, and 0.9 μM) over a TCE of *A. castellanii* (30 μg) at pH 7.0. (100 mM Tris–HCl pH 7, 2 mM CaCl_2_) and pH 5.0 (100 mM sodium acetate pH 5, 2 mM CaCl_2_). For both extracts, 5 mM β-mercaptoethanol and 10 μM Z-Phe-Arg-AMC were added. E-64 (*trans*-Epoxysuccinyl-L-leucylamido(4-guanidino)butane) (100 μM) was used as a positive control for the specific inhibition of the cathepsin L-like CP activities. The experiments were performed in triplicate at least three independent times with similar results. The increase in the Relative Fluorescent Units (RFU) was plotted as a function of time using the Sigma Plot Software (Systat Software Inc. San Jose, CA. USA). The average maximum slope determined the relative proteolytic activity (cathepsin L-like CPs) as RFU/s and then divided by the μg of protein of TCE in each assay (30 μg for *A. castellanii*, and 65 μg for *N. fowleri*). The unit of enzyme activity was defined as RFU/(s * μg) of TCE. The TCE proteolytic activity without any inhibitor was taken as a 100% for the comparison with the TCE proteolytic activity in the presence of different ppTvCP4r concentrations. The values of each data set are shown in Table 1. Statistically significant differences between the means were determined by analysis of variance (ANOVA) using Graph Pad Prism 5.0. The data were analyzed by one-way ANOVA using the Bonferroni method. All pairs of columns in Fig. 1 were compared (*P* < 0.001). The scores with statistically significant differences are indicated with asterisks in the figure. The corresponding *P* values are indicated in the figure legend.
