# Supplementary material for: Dataset of cathepsin L-like CP inhibition of Naegleria fowleri and Acanthamoeba castellanii by ppTvCP4r from Trichomonas vaginalis
Source: Data Brief. 2018 Mar 13;18:404–8. doi: 10.1016/j.dib.2018.03.029 (PMC5996237; doi:10.1016/j.dib.2018.03.029)
Supplement: Supplementary file 1 — Supplementary material. [file mmc1.pdf]

AUTHOR DECLARATION

This manuscript has been submitted exclusively to the *Data in Brief* and implies that it has not been, and will not be published elsewhere including electronically in the same form, in English or in any other language, without the written consent of the copyright-holder. All authors have made substantial contributions in the conception and design of the study, acquisition, analysis and interpretation of data, drafting the article and revising it critically for important intellectual content. Moreover, all authors have read the revised manuscript, accepted responsibility for its contents, have no conflict of interest to declare, have approved its submission for publication and there has been no significant financial support for this work that could have influenced its outcome.

Sincerely,

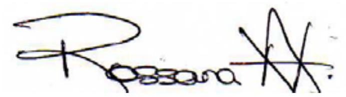

Rossana Arroyo, Ph.D. (on behalf of all authors)
Corresponding author
E-mail: rarroyo@cinvestav.mx
